# Ultrasonic Extraction of Antioxidants from Chinese Sumac (*Rhus typhina* L.) Fruit Using Response Surface Methodology and Their Characterization

**DOI:** 10.3390/molecules19079019

**Published:** 2014-06-27

**Authors:** Jixiang Lai, Huifang Wang, Donghui Wang, Fang Fang, Fengzhong Wang, Tao Wu

**Affiliations:** Institute of Agro-products Processing Science and Technology, Chinese Academy of Agricultural Sciences, Beijing 100193, China

**Keywords:** *Rhus typhina* L*.*, antioxidant, phenolics, pyranoanthocyanin, ultrasonic assisted extraction, response surface methodology

## Abstract

For the first time, response surface methodology (RSM) using a Box-Behnken Design (BBD) was employed to optimize the conditions for ultrasonic assisted extraction (UAE) of antioxidants from Chinese sumac (*Rhus typhina* L.) fruits. Initially, influencing factors such as liquid-solid ratio, duration of ultrasonic assisted extraction, pH range, extraction temperature and ethanol concentration were identified using single-factor experiments. Then, with respect to the three most significant influencing factors, the extraction process focusing on the DPPH· scavenging capacity of antioxidants was optimized using RSM. Results showed that the optimal conditions for antioxidant extraction were 13.03:1 (mL/g) liquid-solid ratio, 16.86 min extraction time and 40.51% (v/v) ethanol, and the desirability was 0.681. The UPLC-ESI-MS analysis results revealed eleven kinds of phenolic compounds, including four major rare anthocyanins, among the antioxidants. All these results suggest that UAE is efficient at extracting antioxidants and has the potential to be used in industry for this purpose.

## 1. Introduction

Phytochemicals with antioxidant capacity are valuable components for industrial food design, and natural plants are an important source of these compounds. Staghorn sumac (*Rhus typhina* L.) is a perennial and flowering shrub that belongs to the genus Rhus. It originally came from Canada and the United States and is widely distributed in the Northern Hemisphere’s subtropical to temperate regions [1]. Its fruit is traditionally used to make a beverage termed “rhus juice” that has served as a drink with medicinal properties for indigenous peoples for centuries [2]. Staghorn sumac extracts have been shown to possess strong antimicrobial and antioxidant activities [3,4]. The antioxidant activities were found by HPLC-MS to be due to the presence of phenolic acids and flavonoids, as well as five major anthocyanins [5]. More recently, two unusual 7-*O*-methylpyranoanthocyano vinylcatechol aglycones, sumadin A and sumadin B, have been identified in staghorn sumac by NMR spectroscopic methods [6]. Staghorn sumac has been extensively cultivated in North China since 1959 and is mainly used for forestation and gardening purposes [7]. Staghorn sumac has often been grown in regions that are not agriculturally viable for other purposes, and it has been used historically for food and medicinal purposes, which suggests there is potential for commercializing the bioactivity of these plants without competing for land used for food production.

One of the most important aspects of the production chain for medicinal plants is the extraction process as this directly influences the quality and quantity of the active compounds produced. Thus, to avoid long processing times, the extraction method and parameters must be optimized. Ultrasonic assisted extraction (UAE) is a simple pretreatment process using ultrasonic waves to effectively accelerate the release of the target compounds into the solvent. Compared with conventional extraction techniques, interest in UAE has increased significantly due to its inherent advantages such as simplified manipulation, significant reduction in energy consumption, lower temperature and higher efficiency [8,9,10]. Response surface methodology (RSM) is an optimization method for experimental processes that is a simple, effective and accurate tool that uses statistical and mathematical techniques.

After an extensive literature search, no previous reports were found on the use of UAE combined with RSM in the extraction of Chinese sumac fruits. Therefore, in this study, UAE and RSM were both employed to optimize the extraction conditions of antioxidants from sumac fruits on the basis of a single factor method. Three influencing factors in the water-ethanol extraction of antioxidants, namely liquid-solid ratio, duration of UAE and ethanol concentration in water, were investigated. The radical scavenging activity of the water-ethanol extract from sumac fruits was studied using an *in vitro* chemical reaction with the stable radical DPPH·. In addition, the antioxidants from the Chinese sumac fruits were identified using UPLC-MS.

## 2. Results and Discussion

### 2.1. Results of Single-Factor Experiments

**Figure 1:**
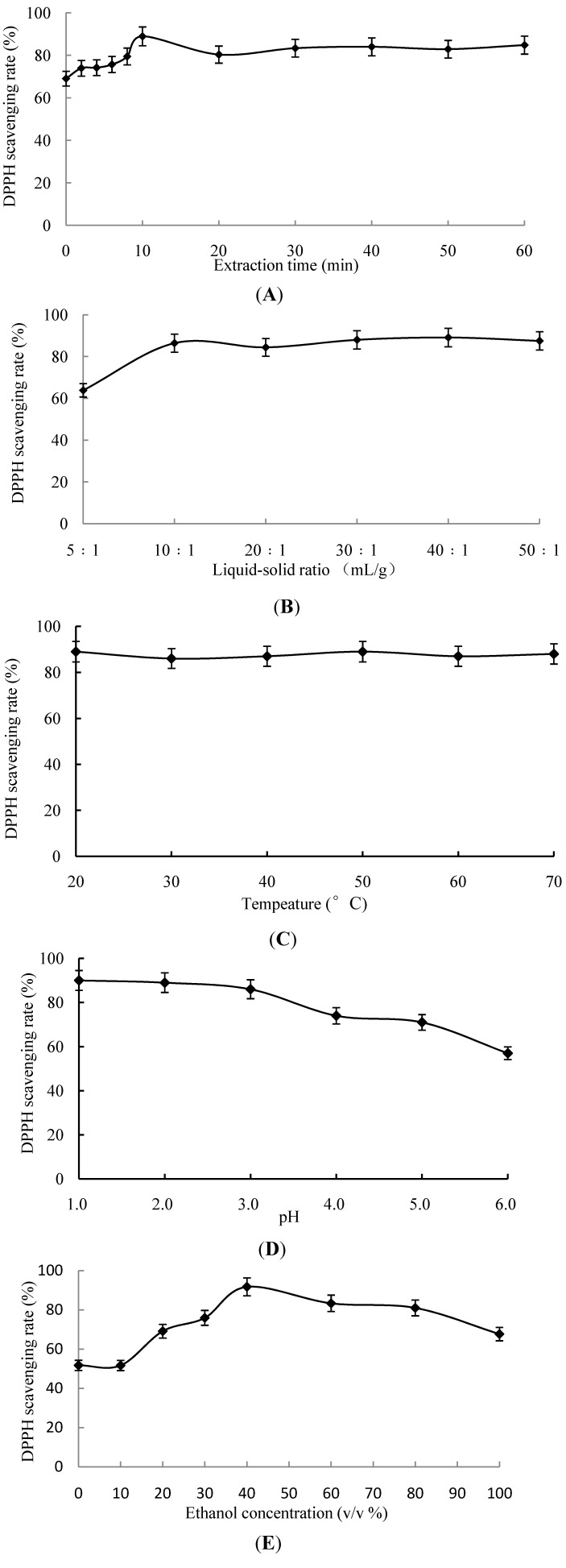
The influence of (**A**) extraction time; (**B**) Liquid-solid ratio; (**C**) Extraction temperature; (**D**) pH of extraction solvent and (**E**) Ethanol concentration on antioxidant activities of extraction.

The influence of extraction time, liquid-solid ratio, extraction temperature, pH and ethanol concentration on antioxidant activities after extraction are shown in Figure 1.

It can be seen from Figure 1A that from 0 to 10 min, the DPPH· scavenging activities increased as extraction time increased, and they reached more than 88% after 10 min before declining to about 83% after 20 min. Between the scavenging activities recorded after extraction times between 20 and 60 min, there were no significant differences (*p* > 0.05). Therefore, it can be seen that the greatest scavenging activity was obtained after extraction for 10 min. This might be due to the available active ingredients being released quickly after the ultrasonic extraction is started and then quickly accumulating in the extraction solvent. Then after 10 min, the greatest free radical scavenging activity was obtained which is when the greatest concentration of active ingredients was in the solvent. The scavenging capacity reached its maximum value at 10 min, and continued extraction for 60 min resulted in a slight decrease in the DPPH· scavenging level. This may be due to the degradation of certain active ingredients during ultrasonic extraction coupled with the emulsification effect of ultrasound influencing the active groups of the active ingredients.

It can be seen from Figure 1B that as the liquid-solid ratio increased from 5:1 mL/g to 10:1 mL/g the scavenging activity increased, and then it remained constant as the ratio increased to 50:1 mL/g. This may be due to the fact that less active ingredients can be extracted when using a small quantity of extraction solvent (liquid-solid ratio = 5:1 mL/g). Increasing the liquid-solid ratio to 10:1 mL/g caused an increase in the scavenging level of the DPPH·. Then, at a certain point, all of the antioxidative substances that could be, had been extracted from the sumac. Hence, the scavenging activity no longer increased when one continues to increase the liquid-solid ratio. Therefore, the most appropriate liquid-solid ratio was determined to be 10:1 mL/g.

The effect of changes in temperature on the sumac extract’s free radical scavenging activities are shown in Figure 1C. It was not possible to observe significant differences (*p* < 0.05) between the antioxidant in the extracts obtained between 20 °C and 70 °C form sumac. Therefore, temperature was not considered to be an influencing factor on the extraction of antioxidants from sumac.

The effect of pH on the extraction of antioxidants from sumac fruits is shown in Figure 1D. The natural pH was 6.0, and when it was adjusted to be 1.0 the extraction of antioxidants increased. The DPPH· scavenging activities obtained using pH = 2.0 and 1.0 ethanol solutions were not significantly different (*p* > 0.05). Due to the strongly acidic extract solvent (pH = 1.0) resulting in processing difficulties and influencing the DPPH radical scavenging activities, the pH = 2.0 was selected as the optimal acidic condition for the extraction.

A marked increase in the DPPH· scavenging rate was observed as the ethanol concentration increased from 10% to 40%, as shown in Figure 1E. The maximum scavenging activity was obtained at a concentration of 40%; however, further increases in the concentration to 100% did not further increase the scavenging level but rather decreased it. The reason for this could be that some antioxidants from sumac were soluble in water while others were soluble in ethanol, and for those soluble in the former the antioxidant activity is greater [11]. This result is in accordance with the results of Shi *et al.* [12], where aqueous ethanol was more efficient than water for extracting phenolics from grape seeds. Therefore, 40% (v/v) was regarded as the optimal ethanol concentration for extraction.

In summary, from all the above results, it can be concluded that extraction temperature was not an influencing factor and that pH = 2.0 was the optimal acidic conditions to be used in the extraction of antioxidants from sumac. The three influencing factors that were most significant were ethanol concentration, extraction time and liquid-solid ratio. The effective experimental ranges selected for the RSM study were ethanol concentration from 20% to 60 % (v/v), extraction time of 5 to 35 min and liquid-solid ratio from 5:1 to 15:1 (mL/g), based on the results of the single-factor experiments.

### 2.2. Results of Response Surface Methodology Experiments

The effect of the three independent variables, namely, ethanol concentration (A), extraction time (B) and liquid-solid ratio (C) on DPPH· scavenging rate (Y) was investigated using a three-factor BBD-RSM experimental setup and the results are shown in Table 1.

The final equation in terms of coded factors was as follows:

DPPH scavenging rate: Y = 91.77 + 1.25A − 1.16B + 2.58C − 6.28A^2^ − 3.17B^2^ − 2.12C^2^ − 1.88AB − 2.20AC − 0.21BC (1)

The significance of the RSM was determined by performing an analysis of variance (ANOVA), and the results are shown in Table 2. As shown in Table 2, the Model F-value of 45.07 implies that the model is significant. The chance that noise could have resulted in a “Model F-Value” this large is 0.01%. Values of “Prob > F” less than 0.05 indicated that the model terms are significant. In this case A, B, C, A^2^, B^2^, C^2^, AB and AC are significant model terms. The “Lack of Fit F-value” of 3.98 implies that the Lack of Fit is not significant relative to the pure error. There is a 10.75% chance that a “Lack of Fit F-value” this large could occur due to noise. Due to the desire to develop a model that fits the data, the fact that the lack of fit is non-significant is good.

**Table 1:** Response surface analysis program and results for sumac extract.

Run	Factor1 A: Ethanol Concentration (%)	Factor2 B: Extraction Time (min)	Factor3 C: Liquid-Solid Ratio	DPPH· Scavenging Rate Y: (%)
1	20	35	10:1	82.03
2	40	20	10:1	91.77
3	60	35	10:1	79.62
4	40	5	15:1	90.43
5	20	5	10:1	81.28
6	60	5	10:1	86.37
7	40	35	15:1	88.38
8	40	20	10:1	92.08
9	20	20	15:1	85.97
10	20	20	5:1	77.10
11	40	20	10:1	92.60
12	40	35	5:1	82.95
13	40	20	10:1	91.47
14	40	20	10:1	90.95
15	60	20	5:1	85.17
16	60	20	15:1	85.25
17	40	5	5:1	84.15

**Table 2:** ANOVA for Response Surface Quadratic Model: Analysis of variance table [Partial sum of squares].

Source	Sum of Squares	DF	Mean Square	F Value	Prob > F	significant
Model	357.60	9	39.73	45.07	<0.0001	significant
A	12.58	1	12.58	14.26	0.0069	
B	10.70	1	10.70	12.13	0.0102	
C	53.35	1	53.35	60.52	0.0001	
A2	165.90	1	165.90	188.19	<0.0001	
B2	42.36	1	42.36	48.06	0.0002	
C2	19.00	1	19.00	21.56	0.0024	
AB	14.06	1	14.06	15.95	0.0052	
AC	19.32	1	19.32	21.91	0.0023	
BC	0.18	1	0.18	0.20	0.6645	
Residual	6.17	7	0.88			
Lack of Fit	4.62	3	1.54	3.98	0.1075	not significant
Pure Error	1.55	4	0.39			
Cor Total	363.77	16				

The analysis of the RSM model is shown in Table 3. The “Pred R-Squared” of 0.7900 is in reasonable agreement with the “Adj R-Squared” of 0.9612. The signal to noise ratio is measured by the “Adeq Precision”, where a value that is greater than 4 is needed. Therefore, the ratio of 20.046 indicates an adequate signal, which means this model can be used to navigate the design space.

**Table 3:** Analysis of RSM model.

Standard Deviation	0.94	R-Squared	0.9830
Mean	86.33	Adj R-Squared	0.9612
Coefficient Of Variation	1.09	Pred R-Squared	0.7900
PRESS	76.40	Adeq Precision	20.046

**Figure 2:**
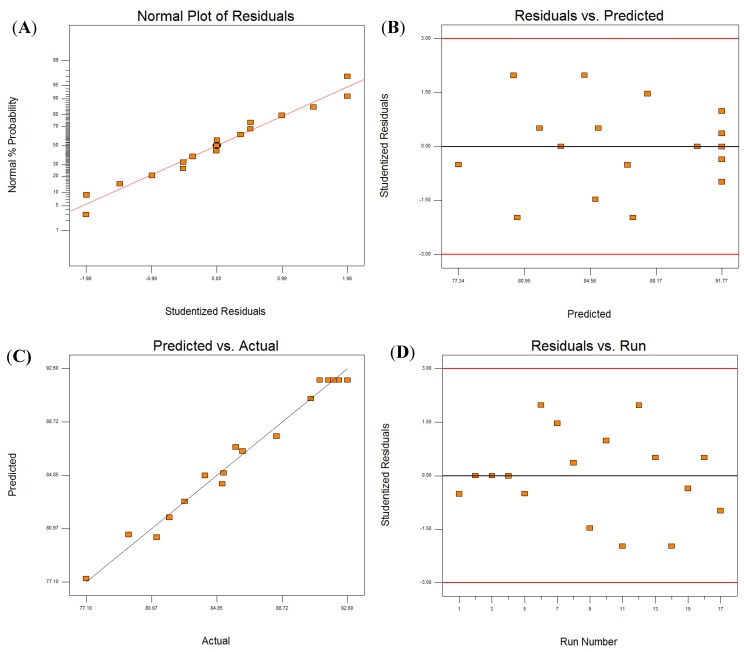
Analysis of RSM model: (**A**) Normal Plot of Residuals; (**B**) Residuals *vs*. Predicted; (**C**) Predicted *vs*. Actual; (**D**) Residuals *vs*. Run.

Figure 2 shows that the Normal Plot of Residuals (A) and Predicted *vs*. Actual (C) were both straight lines; while, the Residuals *vs*. Predicted (B) and Residuals *vs*. Run (D) were scattered randomly. From the results it can therefore be seen that the model is suitable for use and can be used to identify the optimal extraction parameters.

**Figure 3:**
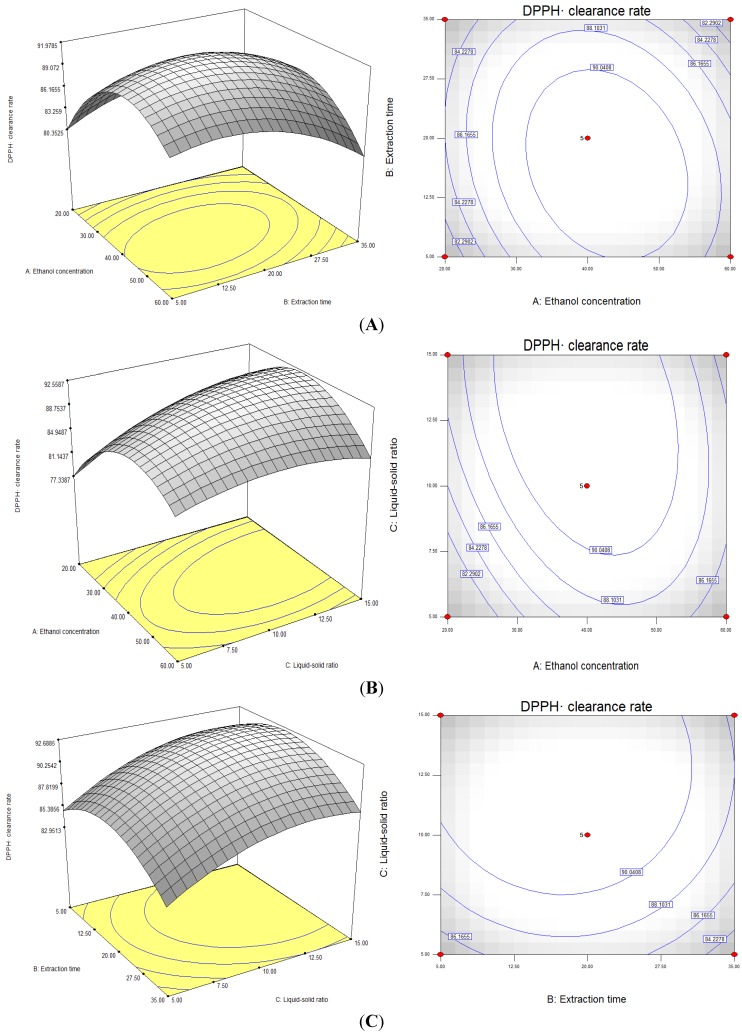
Response surfaces and contour plots showing: (**A**) the effects of ethanol concentration and extraction time on DPPH· scavenging rate Y = (A, B); (**B**) the effects of ethanol concentration and liquid-solid ratio on DPPH· scavenging rate Y = (A, C); (**C**) the effects of extraction time and liquid-solid ratio on DPPH· scavenging rate Y = (B, C).

The results shown in Figure 3 imply that ethanol concentration, extraction time and liquid-solid ratio were significant model terms in this case. The optimal solutions analyzed using Design Expert 7.1 were as follows: ethanol concentration, 40.51% (v/v); extraction time, 16.86min; liquid-solid ratio, 13.03:1 mL/g); DPPH· scavenging rate, 92.70%; and desirability, 0.681. Under these conditions, the measured actual DPPH· scavenging rate can reach 92.50% ± 0.63%, which is similar to the theoretical prediction.

### 2.3. Characterization of Antioxidants by UPLC-MS

**Figure 4:**
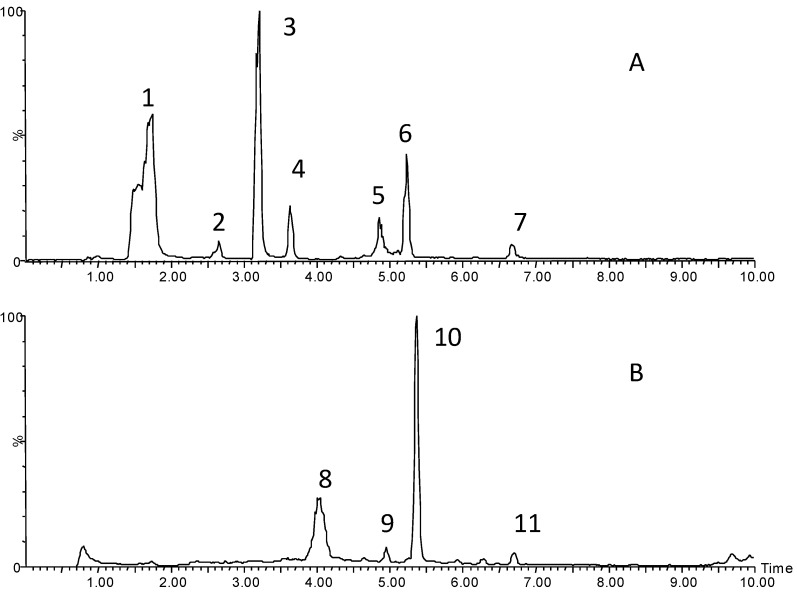
UPLC-MS total Ion chromatographic profile of Chinese sumac: (**A**) electrospray ion source (ESI) operating in negative mode; (**B**) ESI operating in positive mode.

MS analysis for non-anthocyanin phenolics was carried out using an electrospray ionisation source in the negative mode (Figure 4A), while, MS analysis for anthocyanins was performed in the positive mode (Figure 4B). A total of 11 phenolic compounds were tentatively identified (Table 4).

**Table 4:** Peak assignment, retention time (Rt) and mass spectral data of compounds detected in Chinese sumac fruits.

Peak	Rt (min)	Primary *m/z* Fragment	Proposed Identity
1	1.74	[M^−^] 169.0	Gallic acid
2	2.66	[M^−^] 341.0	Caffeic acid -*O*-hexose
3	3.20	[M^−^] 341.0	Caffeic acid -*O*-hexose
4	3.63	[M^−^] 341.0	Caffeic acid -*O*-hexose
5	4.85	[M^−^] 301.0	Ellagic acid
6	5.23	[M^−^] 447.0	Quercetin-3-rhamnoside
7	6.67	[M^−^] 301.0	Quercetin
8	4.04	[M^+^] 615.0	7-*O*-Methylcyanidin-3-*O*-(2ꞌꞌgalloyl)-β-d-galactoside
9	4.96	[M^+^] 925.0	7-*O*-Methyldelphinidin-3-*O*-(2ꞌꞌꞌgalloyl)-β-d-galactoside-4-vinyl- catechol-3ꞌꞌ-*O*-glucoside
10	5.36	[M^+^] 909.2	7-*O*-Methylcyanidin-3-*O*-(2ꞌꞌꞌgalloyl)-β-d-galactoside-4-vinyl- catechol-3ꞌꞌ-*O*-glucoside
11	6.71	[M^+^] 747.0	7-*O*-Methylcyanidin-3-*O*-(2ꞌꞌꞌgalloyl)-β-d-galactopyranosyl- 4-vinylcatechol

It was found that the composition of the Chinese sumac reported here was similar to that of according to previously published information [6]. Peaks 2 and 4 also had a deprotonated molecular ion of *m/z* 341.0 [M]^−^, and were tentatively identified as two isomers of glucocaffeic acid. Peaks 4, 5 and 6 showed deprotonated molecular ions of *m/z* 301.0, *m/z* 447.0 and *m/z* 301.0 respectively, and were identified as ellagic acid, quercetin-3-rhamnoside and quercetin, respectively. From the UPLC total ion chromatogram (Figure 4B), it was shown that Chinese sumac had a rich assortment of anthocyanin compounds, as seen by four major visible peaks. Peak 8 had a molecular ion [M]^+^ at 615.0, and was identified as 7-*O*-methylcyanidin-3-*O*-(2ꞌꞌ galloyl)- β-d-galactoside; this is a rare 7-*O*-methyl cyanidin compound as proven from the NMR spectrum [6]. As shown by the molecular ion [M]^+^ at 925.0 *m/z*, there is another unusual anthocyanidin with an unusual aglycone at [M]^+^ = 433 Da [5] that was recently identified as 7-*O*-methyl-delphinidin-3-*O*-(2ꞌꞌꞌgalloyl)-β-d-galactopyranoside-4-vinyl- catechol-3ꞌꞌ-*O*-β-d-glucopyranoside by its NMR spectrum [6]. Peak 10 had [M]^+^ at 909.2 and a cryptic pyranoanthocyanin structure, which corresponded to 7-*O*-methylcyanidin-3-*O*-(2ꞌꞌꞌgalloyl)-β-d- galactopyranoside-4-vinylcatechol-3ꞌꞌ-*O*-β-d-glucopyranoside [6]. Peak 11 with 747.0 *m/z* had one hexose moiety (162 amu) less than the fragment of peak 10 and was also found to have an unusual aglycone with [M]^+^ = 433 Da. Peak 10 was tentatively identified as 7-*O*-methylcyanidin-3-*O*- (2ꞌꞌꞌgalloyl)-β-d-galactopyranosyl-4-vinylcatechol, which is proposed for the first time. Although it has been found that the degradation of active ingredients may occur during application of UAE in food processing [13,14], the results showed that the UPLC-MS profile of the major ingredients in the extract was the same as our previous studies [5,6], which indicated that the components of the sumac fruit extract were stable.

## 3. Experimental

### 3.1. Materials and Reagents

Staghorn sumac fruits were harvested in September 2013 in Beijing, China. 1,1-Diphenyl- 2-picrylhydrazyl (DPPH) radical reagent was obtained from Sigma (Shanghai, China). All other chemical reagents used were of analytical grade.

### 3.2. Antioxidant Extraction

Gentle rubbing by hand was used to collect the staghorn sumac fruits from the staghorn plant. Then about 200 g of the collected fruits were freeze dried (Bulk Tray Dryer, Labconco, Kansas City, MO, USA) and ground with a blender, before being filtered through 40 mesh sieve. The powder (1.0 g) was then mixed with ethanol of different concentrations to form the required liquid-solid ratios. Then the mixture was adjusted to different pH concentrations and extracted using the JCX-100G ultrasonic instrument (Hengsheng Ultrasonic Machinery Corporation, Jining, China). The extract was centrifuged (Sigma 4K-15, Goettingen, Germany) at 10,000 rpm for 10 min at room temperature, after which the supernatant was filtered through a 0.2-μm PTFE membrane filter (VWR International, Mississauga, ON, Canada) in preparation for spectrophotometric and UPLC-MS analyses. All procedures were conducted under reduced light levels, and all experiments were conducted three times and an average was obtained.

### 3.3. Determination of Antioxidant Capacity of Extract

The DPPH radical scavenging capacity assay was based on a previously described method [15] with some modifications. Briefly, an ethanolic solution of DPPH (100 μL, 0.20 mM) was mixed with the solution of the extract (100 μL) and left to stand for 30 min at 37 °C. The absorbance was recorded at 517 nm using a Spectra Max 190 absorbance plate reader (Molecular Device, Sunnyvale, Calif, USA). All samples were diluted 200 times before analysis. The radical scavenging activity of the extracts was calculated as follows:

Percent Scavenging (%) = [A_0_ − (A_1_ − A_s_)]/A_0_ × 100% (2)

where A_0_ is the absorbance of DPPH alone, A_1_ is the absorbance of DPPH + extract and A_S_ is the absorbance of the extract only. All samples were tested in triplicate.

### 3.4. Single-Factor Experiments

Initially, the appropriate ranges and influencing factors were determined in a preliminary study so that they could be used to design the main experiments. Many factors including extraction time, composition of solvent and solvent to solid ratio, could affect the UAE extraction efficiency. All of these factors were evaluated to identify the most influential factors when trying to obtain the maximum possible extraction of the antioxidant compounds when using UAE.

### 3.5. Response Surface Methodology Experiments

RSM was employed to establish the optimum conditions for extracting antioxidants from Chinese sumac [16,17]. The effect of three independent variables, namely, ethanol concentration (20%–60%, v/v), extraction time (5–35min) and liquid-solid ratio (5:1–15:1, mL/g), on DPPH· scavenging activity was investigated using a three-factor BBD (Box-Behnken Design)-RSM experimental run to determine the optimal parameters of the extraction process. Experimental factors and levels are shown in Table 5.

**Table 5:** Factors and levels of RSM.

Levels	Independent Variables		
	A: Ethanol Concentration (%)	B : Time (min)	C: Liquid-Solid Ratio (mL/g)
−1	20	5	5:1
0	40	20	10:1
1	60	35	15:1

### 3.6. UPLC-MS

The chromatographic analyses of the extracts were performed on a UPLC Acquity chromatograph coupled with a TQD Acquity mass spectrometer (Micromass-Waters, Manchester, England), which had an electrospray ionization (ESI) source. A C18 BEH Waters Acquity column (2.1 mm × 100 mm, 1.7 μm particle size) was used [18]. Solvents A and B were methanol and water with 0.2% formic acid, respectively. The flow rate was 0.3 mL/min and 2 μL of samples were injected; with a linear gradient starting at 10% methanol, 0–1 min, 10%–20%, 1–3 min, 20%–45%, 3–8 min, 45%–50%, 8–10 min, 50%–90%, held until 12 min and then returned to the initial conditions, followed by column re-equilibration. The ESI was used with the following conditions for the positive mode: capillary 4.00 kV, cone 30 V, source temperature 120 °C and desolvation temperature 350 °C. While, in the negative ion mode: capillary −3.00 kV, cone −30 V, source temperature 150 °C, desolvation temperature 350 °C and collision energy 30 V. Data was obtained from between 100 and 1000 *m/z*.

### 3.7. Statistical Methods

The analyses of the data were done using SPSS v19.0 statistical package (IBM Corporation, New York, NY, USA). The experimental data were subjected to χ 2 tests, and *p* < 0.05 was considered to be a significant difference. The analyses of the RSM data were done using Design-Expert 7.1 (Stat-Ease, Inc. Minneapolis, MN, USA).

## 4. Conclusions

The study represents the first report on the feasibility of ultrasound-assisted extraction for the maximizing recovery of antioxidants from Chinese sumac. RSM was used to investigate the main and interaction effects of important independent variables for extraction of antioxidants on the basis of single-factor experiments. Ultrasound-assisted extraction with acidified ethanol was proved to be efficient technique for easy and rapid isolation of high yields of antioxidants from Chinese sumac at room temperature (20 °C). The optimal conditions for antioxidants extraction were 13.03:1 (mL/g) liquid-solid ratio, 16.86 min extraction time and 40.51% (v/v) ethanol, and the desirability was 0.681. In addition, a total of 11 phytochemicals including phenolic acids, flavonoids and anthocyanins were identified from the antioxidants by UPLC-MS. Pyranoanthocyanins are thought to contribute to the orange hues observed during wine maturation and aging and have higher antioxidant potential [19]. Therefore, sumac can be regarded as a new resource of pyranoanthocyanins in the production of natural functional foods, food additives and dietary supplements.

## References

[1] Rayne S., Mazza G. (2007). Biological activities of extracts from Sumac (*Rhus spp.*): A review. Plant Food. Hum. Nutr..

[2] Foster S., James A.D., Peterson R.T. (2000). A Field Guide to Medicinal Plants and Herbs of Eastern and Central North America.

[3] Kossah R., Zhang H., Chen W. (2011). Antimicrobial and antioxidant activities of Chinese sumac (*Rhus typhina* L.) fruit extract. Food Control.

[4] McCune L., Johns T. (2002). Antioxidant activity in medicinal plants associated with the symptoms of diabetes mellitus used by the indigenous peoples of the North American boreal forest. J. Ethnopharmacol..

[5] Wu T., McCallum J.L., Wang S.N., Liu R.H., Zhu H.H., Tsao R. (2013). Evaluation of antioxidant activities and chemical characterisation of staghorn sumac fruit (*Rhus hirta* L.). Food Chem..

[6] Kirby C., Wu T., Tsao R., McCallum J. (2013). Isolation and structural characterization of unusual pyranoanthocyanins and related anthocyanins from Staghorn sumac (*Rhus typhina* L.) via UPLC-ESI-MS, H^−1^, C^−13^, and 2D NMR spectroscopy. Phytochemistry.

[7] Wang G., Jiang G., Yu S., Li Y., Liu H. (2008). Invasion possibility and potential effects of *Rhus typhina* on Beijing municipality. J. Integr. Plant Biol..

[8] Lai J., Xin C., Zhao Y., Feng B., He C., Dong Y., Fang Y., Wei S. (2013). Optimization of ultrasonic assisted extraction of antioxidants from Black Soybean (*Glycine max var*) sprouts using response surface methodology. Molecules.

[9] Rajendran V., Karuppan M. (2012). Ultrasound-assisted alkaline pretreatment of sugarcane bagasse for fermentable sugar production: Optimization through response surface methodology. Bioresource Technol..

[10] Chemat F., Zill-e-Huma, Khan M. (2011). Applications of ultrasound in food technology: Processing, preservation and extraction: A review. Ultrason. Sonochem..

[11] Bursal E., Köksal E. (2011). Evaluation of reducing power and radical scavenging activities of water and ethanol extracts from sumac (*Rhus coriaria* L.). Food Res. Int..

[12] Shi J., Yu J., Pohorly J., Young J., Bryan M., Wu Y. (2003). Optimization of the extraction of polyphenols from grape seed meal by aqueous ethanol solution. J. Food Agric. Environ..

[13] Daniella P., Anne-Sylvie F., Farid C. (2013). Degradation during application of ultrasound in food processing: A Review. Food Control.

[14] Daniella P., Grégory D., Anne-Sylvie F., Antal R., Christian G., Farid C. (2012). Degradation of edible oil during food processing by ultrasound: Electron paramagnetic resonance, physicochemical, and sensory appreciation. J. Agric. Food Chem..

[15] Wu T., Yan J., Liu R., Marcone M., Aisa H., Tsao R. (2012). Optimization of microwave-assisted extraction of phenolics from potato and its downstream waste using orthogonal array design. Food Chem..

[16] Mehrnoush A., Mustafa S., Sarker M., Yazid A. (2011). Optimization of the conditions for extraction of serine protease from Kesinai Plant (*Streblus asper*) leaves using response surface methodology. Molecules.

[17] Liu Y., Wei S., Liao M. (2013). Optimization of ultrasonic extraction of phenolic compounds from Euryale ferox seed shells using response surface methodology. Ind. Crop. Prod..

[18] Hector H., Felipe M., Fábio C., Antonia Q., Afonso D. (2013). Antioxidant, antimicrobial activities and characterization of phenolic compounds from buriti (*Mauritia flexuosa* L. f.) by UPLC-ESI-MS/MS. Food Res. Int..

[19] Azevedo J., Oliveira J., Cruz L., Teixeira N., Brás N., de Freitas V., Mateus N. (2014). Antioxidant Features of Red Wine Pyranoanthocyanins: Experimental and Theoretical Approaches. J. Agric. Food Chem..

